# Cosmeceutical and Dermatological Potential of Shiitake Mushroom (*Lentinula edodes*) Extract

**DOI:** 10.1111/jocd.70724

**Published:** 2026-02-08

**Authors:** Sibel Dikmen Kucuk, Paul Jabet, Anthony Groso, Guillaume Collet, Richard Daniellou, Beste Karadeniz

**Affiliations:** ^1^ Coordination Office of Specialization in Environment and Health Technologies Duzce University Duzce Turkey; ^2^ Chaire de Cosmétologie, Institute of AgroParisTech Orleans France

**Keywords:** antiaging activity, elastase and collagenase inhibition, HaCaT keratinocytes, *Lentinula edodes*, topical cream formulation

## Abstract

**Background:**

Shiitake mushroom (*Lentinula edodes*) contains polysaccharides, phenolics, and β‐glucans known for their antioxidant and nutraceutical properties; however, its dermatological applications remain limited.

**Objective:**

This study aimed to evaluate the antioxidant potential, enzyme inhibition activities, and cytotoxicity of freeze‐dried Shiitake extract and to develop and characterize a topical cream formulation containing the extract for potential cosmetical and dermatological use.

**Methods:**

Shiitake mushrooms were extracted with 75% ethanol and freeze‐dried. Antioxidant capacity was assessed using a total antioxidant assay. Elastase, collagenase, and tyrosinase inhibition activities were evaluated using fluorometric and spectrophotometric assays. Cytotoxicity was tested on HaCaT human keratinocytes using the AlamarBlue assay. A topical cream formulation containing the shiitake extract was prepared, and its pH, viscosity, density, and stability were assessed through accelerated stability tests.

**Results:**

The extract showed moderate antioxidant capacity (107 μM Trolox equivalent). It inhibited elastase activity by 51.99% and induced a concentration‐dependent suppression of collagenase activity, reducing measurable enzyme activity to levels below the detection limit of the assay at higher concentrations. No significant tyrosinase inhibition was observed. Cytotoxicity assays confirmed that the extract was non‐toxic, maintaining > 90% HaCaT viability across all tested concentrations. The formulated cream exhibited skin‐compatible pH (≈5.5), suitable viscosity, homogeneous texture, and remained stable without phase separation or microbial growth under accelerated aging conditions.

**Conclusion:**

Shiitake extract appears to be a safe and naturally derived bioactive ingredient, exhibiting notable elastase and collagenase inhibitory activities and demonstrating promising potential for incorporation into anti‐aging and nutraceutical skin‐care formulations.

## 1 Introduction

Medicinal mushrooms have long been considered traditional medicines due to their nutritional values and various metabolic components, such as proteins, oxalic acid and peptides, terpenes, steroids, anthraquinone, phenolic acid, and benzoic acid [1, 2]. Their antiviral, antibacterial, antiparasitic, antifungal, wound healing, anticancer, immunomodulatory, antioxidant, radical scavenging, detoxification, hepatoprotective, cardiovascular, antihypercholesterolemia, and antidiabetic effects have been reported in many studies and have attracted significant interest for further investigation in the nutraceutical and pharmaceutical fields [3, 4]. One of the five most cultivated medicinal edible mushrooms in the world is *Lentinus edodes*, commonly known as the Shiitake mushroom. Shiitake has now gained increasing market and popularity worldwide, particularly in Asia, Europe, and North America, with advances in research demonstrating its medicinal properties [5, 6]. Shiitake mushrooms contain almost all the essential amino acids needed by the human body [7] and are rich in unsaturated fatty acids [8]. Additionally, extracts from shiitake mushrooms have been found to contain polysaccharides [9], glycoproteins [10], phenolic compounds [11], terpenoids [12], minerals, and vitamins [13]. Numerous recent studies have demonstrated its medicinal properties, including antitumor, antimicrobial, liver function‐improving, and cholesterol‐lowering activity [14]. However, studies examining its effects in dermatological and cosmeceutical products are limited.

Cosmeceuticals are skin care products that blend cosmetic and pharmaceutical benefits. They refer to the application of metal‐containing substances with drug‐like properties to improve skin appearance, reduce hyperpigmentation, and provide anti‐aging benefits [15]. These two categories, along with nutricosmetics, which act from the inside out, have gained popularity among consumers worldwide due to their effective approaches to skin health [16], and new natural and organic chemicals that can provide competitive skin care are sought [17, 18]. The cosmeceutical industry, which has introduced a variety of substances derived from natural sources, includes antioxidants, collagenase, elastase, and tyrosinase inhibitors and the most commonly used bioactive compounds [19].

Anti‐aging cosmeceuticals are produced to improve or maintain the body's maintenance and repair processes. Aging is a progressive process that causes dysfunction and a decrease in reserve capacity in all body organs [20]. The ultimate pathway of all aging mechanisms appears to be the same, including the degradation of the collagen and elastin network. Collagen is the most important component of the skin's extracellular space and is responsible for maintaining skin elasticity or resilience. UV rays cause collagen destruction, leading to aging [21]. The components of the skin's extracellular environment (hyaluronic acid, collagen, and elastin) decrease as people age, leading to decreased strength and elasticity and the formation of wrinkles [22]. Collagenases are a category of matrix metalloproteinases that can break the peptide bonds in naturally occurring collagen fibrils. Fungi improve the natural skin barrier by increasing collagen formation, nourishing elasticity, and increasing moisture levels. In a study in which dermal fibroblast cells were treated with an extract from *Tricholoma matsutake* for 72 h, the extract was found to exhibit significant anti‐elastase and anti‐collagenase activities [23]. As a result, it was concluded that natural phenolic compounds found in mushrooms can be used in the treatment of skin aging, restoring skin resistance, stimulating collagen formation, increasing moisture content, and in anti‐aging cosmeceutical skin products as inhibitors of tyrosinase, elastase, and collagenase enzymes. They are also promising candidates in the treatment of hyperpigmentation and skin lightening effects due to their antioxidant properties against free radicals [19, 24].

This study aimed to investigate the bioactive potential of *Lentinula edodes* (shiitake mushroom), which, in addition to its high nutritional value, contains various components beneficial for skin and overall health—such as β‐glucans, unsaturated fatty acids, polysaccharides, polyphenols, phenolic compounds, amino acids, minerals, and vitamins. In addition to evaluating its antioxidant capacity, the freeze‐dried shiitake extract was analyzed for elastase and collagenase enzyme inhibition, as well as tyrosinase activity, to assess its potential effects on skin aging and melanin formation. Furthermore, an anti‐aging night cream formulation incorporating shiitake mushroom extract was developed, and its physicochemical properties were characterized. Therefore, this study was designed to explore the cosmetic potential of shiitake mushroom extract by linking its biochemical properties with its applicability in topical formulations, providing a scientific basis for its use in natural skin care products.

## 2 Materials and Methods

### 2.1 Materials

Shiitake mushrooms were purchased from the Agricultural Waste Industrial Recycling Research and Application Center of Düzce University, where they were cultivated on hazelnut pruning residues as a lignocellulosic substrate for subsequent extraction and bioactivity analyses. Other ingredients used in topical cream formulation were purchased from cosmetic raw material suppliers.

### 2.2 Preparation of Freeze‐Dried Shiitake Mushroom Extracts

60 g of shiitake mushroom were ground using a high‐power blender. The ground shiitake mushrooms were then washed in petroleum ether to remove their oils. To minimize the moisture content of the washed shiitake mushrooms evenly, they were dried again in a hot air oven at 35°C for 16 h. The dried shiitake mushrooms were mixed with 75% ethanol (1:10 w/w) and placed in an ultrasonic bath at 40°C for 30 min. Following this process, they were placed in an orbital shaker water bath at 40°C for 1 day. An evaporator was used to remove ethanol from the resulting solution, and then the lyophilization process was carried out in a Vacuum Freeze Dryer (LABFREEZ FD‐10F‐RE) between −50°C and 40°C. As a result, a light brown colored extract was obtained and stored in the refrigerator at +4°C until used. For enzymatic and antioxidant assays, a stock solution of the freeze‐dried extract was prepared at a concentration of 20 mg/mL in dimethyl sulfoxide (DMSO), followed by incubation at 37°C for 5 h with shaking. After centrifugation at 10 000 g, the clear fraction was collected and used for subsequent analyses.

### 2.3 Antioxidant Activity Assay

The total antioxidant capacity (TAC) of the Shiitake mushroom (*Lentinula edodes*) extract was assessed using the Antioxidant Assay Kit (MAK334‐1KT; Merck, Germany). In this assay, antioxidants present in the extract reduce Cu^2+^ ions to Cu^+^, which then reacts with the kit's dye to produce a colored complex. The intensity of the color reflects the overall antioxidant capacity of the sample [25]. Measurements were carried out according to the manufacturer's instructions using a UV–visible spectrophotometer at 570 nm. Trolox was employed as the standard, and results were expressed as μM Trolox equivalents (μM TE). All analyses were performed in triplicate.

### 2.4 Elastase Inhibition Assay

Elastase inhibitory activity was determined using the EnzCheck Elastase Assay Kit (Invitrogen, E12056), following the manufacturer's protocol. The assay is based on the enzymatic cleavage of a synthetic peptide substrate labeled with a fluorophore and quencher pair, which upon hydrolysis releases a fluorescent product proportional to elastase activity. Various concentrations of the Shiitake mushroom extract were tested, ranging from pure extract to serial dilutions down to zero concentration. All extract concentrations are expressed in μg/mL for consistency. Fluorescence intensity was measured using a microplate reader (excitation: 505 nm, emission: 513 nm). The inhibitory activity (%) was calculated relative to the control sample (0% concentration), which was considered to represent 100% elastase activity.

*N‐Methoxysuccinyl‐Ala‐Ala‐Pro‐Val‐chloromethyl ketone* (Sigma‐Aldrich, St. Louis, MO, USA) was used as a reference inhibitor.

### 2.5 Collagenase Inhibition Assay

Collagenase inhibitory activity was measured using the EnzChek Gelatinase/Collagenase Assay Kit (Invitrogen, E12055) following the manufacturer's instructions. The assay employs a highly quenched fluorescein‐conjugated DQ‐gelatin substrate that yields fluorescent fragments upon proteolytic cleavage by gelatinases/collagenases. Fluorescence was measured on a microplate reader (excitation ≈ 495 nm, emission ≈ 515 nm). Various concentrations of the Shiitake mushroom extract were tested, ranging from pure extract to serial dilutions down to zero concentration. All extract concentrations are expressed in μg/mL for consistency. The inhibitory activity (%) was calculated relative to the control sample (0% concentration), which was considered to represent 100% collagenase activity. *Phenantroline* (Sigma‐Aldrich, CAS 5144‐89‐8) was used as a reference inhibitor.

### 2.6 Tyrosinase Inhibition Assay

The tyrosinase inhibitory activity of shiitake mushroom extract was assessed using human tyrosinase (*hs*TYR) obtained from MNT‐1 melanoma cell lysates. This assay is based on the enzymatic oxidation of L‐DOPA to L‐DOPA‐quinone by *hs*TYR, which can be monitored spectrophotometrically through melanin production. A decrease in absorbance at 600 nm indicates that the extract suppresses tyrosinase activity.

MNT‐1 cells were dissociated using 0.05% trypsin–EDTA, washed with PBS (1X), and lysed in PBS containing 1% Triton X‐100. The supernatant was transferred to 96‐well plates, and samples of shiitake extract prepared in DMSO were added at increasing concentrations to achieve a final DMSO concentration of 5% in all wells. After addition of 4 mM L‐DOPA, the reaction mixtures were incubated at 37°C for 4 h. Melanin formation was measured using a UV–visible spectrophotometer, and the inhibitory effect was expressed as percent inhibition relative to the untreated control [26]. All experiments were performed in triplicate.

### 2.7 Cytotoxicity and Determination of Dose Limit

HaCaT human keratinocyte cells were plated at a density of 5 × 10^3^ cells per well in 96‐well plates and allowed to adhere for 24 h at 37°C under 5% CO_2_. The cells were subsequently treated with fresh medium containing varying concentrations of shiitake extract. After 24 h of exposure, cell viability was assessed using the AlamarBlue assay (Invitrogen). This assay relies on the reduction of resazurin to the fluorescent resorufin by metabolically active cells, with higher fluorescence indicating greater cell viability. Fluorescence readings were obtained using a Tecan Infinite M Plex plate reader (excitation: 560 ± 9 nm; emission: 590 ± 20 nm). Cell viability percentages were calculated relative to untreated controls, and the safe concentration range of shiitake extract for subsequent experiments was determined based on these results.

### 2.8 Preparation and Characterization of a Topical Skin Cream Containing Shiitake Extract

The formula table shown in Table 1 was used to prepare the topical face cream formulation. The topical Shiitake extract cream was prepared using a phase‐based emulsification method. Glycerin and xanthan gum were first weighed and mixed, then gradually dispersed into distilled water under continuous stirring to form the aqueous phase. The oil‐phase components were weighed separately in another beaker. Both phases were heated in a water bath (or on a hot plate) for 10–15 min until all solid ingredients were completely melted, with the temperature carefully monitored to reach 70°C–75°C. Once the desired temperature was attained, the oil phase was slowly added into the aqueous phase with continuous stirring to form a uniform emulsion. The mixture was stirred intermittently for 10–15 min while cooling to ensure homogeneity. When the temperature dropped below 40°C, Phase C ingredients were incorporated, followed by Phase D at below 30°C, with gentle mixing after each addition. The final pH of the cream was measured and the finished cream was then transferred into suitable containers, labeled, and stored at +4°C. The pH of the resulting gel was measured with a pH meter (Hanna HI98‐192), its density was measured with a density kit (Precisa 350‐8636), and its viscosity was measured with a viscometer (Fungilab Alpha R).

**TABLE 1:** Formulation of topical shiitake extract cream.

Phase	Function	Component
A	Thickener, Stabilizer	Xanthan Gum
A	Humectant	Glycerin
A	Solvent	Aqua
A	Active	Shiitake Extract
B	Emulsifier	Cetearyl Olivate, Sorbitan Olivate
B	Thickener, Stabilizer	Cetyl alcohol
B	Emollient	Coco Caprylate/Caprate
B	Emollient	Caprylic/Capric Triglyceride
B	Occlusive	Shea butter
C	Antioxidant	Tocopheryl acetate
C	Preservative	Glyceryl Caprylate (And) Glyceryl Undecylenate
D	Fragrance	Ylang‐ylang essential oil
D	Fragrance	Cedar essential oil

### 2.9 Stability Analysis of Topical Skin Cream

Stability analysis of the topical cream obtained from shiitake extract was performed using a stability cabinet device (JSDS‐300C, South Korea). Aging was applied to simulate a 2‐year shelf life. After 24 h at −24°C, 1 week at −5°C, 4 months at room temperature (25°C) and in the dark, and 3 months at +45°C, controls were performed at the 1st week, 1st month, 2nd month and 3rd month. In addition, controls were made after exposure to temperature changes for 6 weeks, at −5°C for 24 h, then +25°C for 24 h. Controls were also made after centrifugation at −25°C, 10 000 rpm for 10 min and 3000 rpm for 30 min [27].

### 2.10 Statistical Analysis

All experiments were performed in triplicate (*n* = 3), and results are presented as mean ± standard deviation (SD). Considering the exploratory nature of the study and its primary aim of preliminary bioactivity screening and formulation characterization, descriptive statistical analysis was applied. Therefore, no inferential statistical comparisons were performed. IC_50_ values were not calculated, as the study was designed as an exploratory screening and the obtained dose–response data were not intended for precise inhibitory constant determination.

## 3 Results

### 3.1 Antioxidant Activity

**FIGURE 1:**
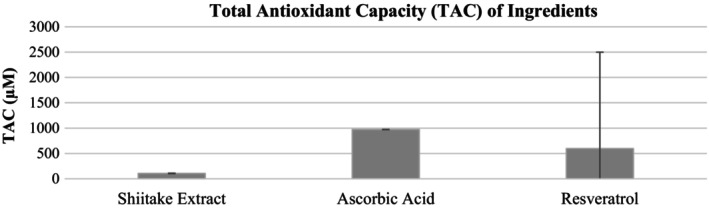
Comparison of the total antioxidant capacity (TAC) among Shiitake extract, ascorbic acid, and resveratrol.

The antioxidant capacity of Shiitake extract was determined to be 107 μM Trolox equivalent (TE) and is shown in Figure 1. Under the same experimental conditions, resveratrol and ascorbic acid showed higher antioxidant activity, corresponding to 596 and 973 μM TE, respectively. These results indicate that the radical‐scavenging capacity of the Shiitake extract is relatively low compared to the standard antioxidants tested, but it can be considered to have moderate antioxidant activity due to its complex composition of phenolic and bioactive compounds.

### 3.2 Elastase Inhibition

**FIGURE 2:**
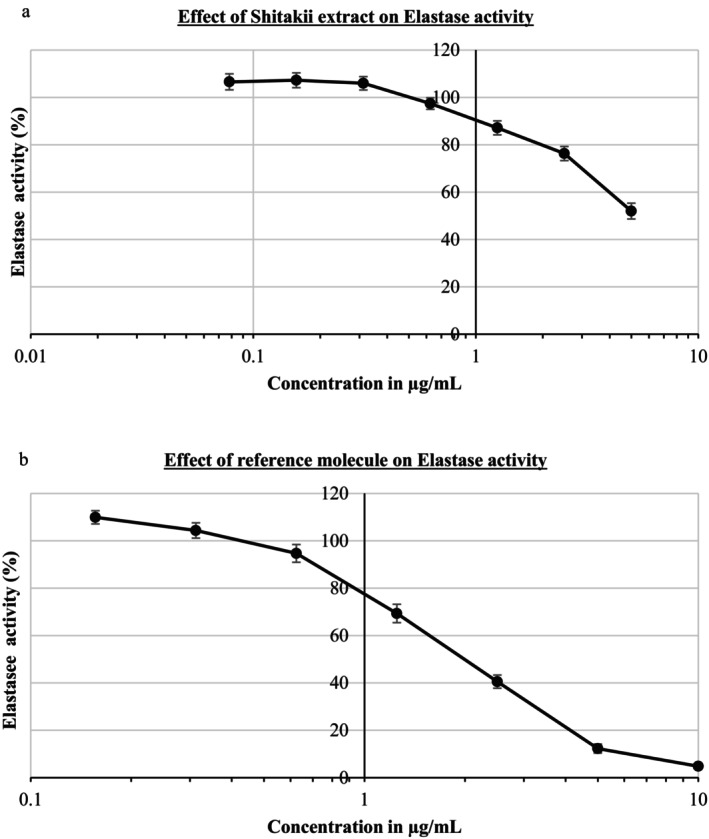
Elastase activity of shiitake extract (a) and reference molecule (b).

The elastase inhibitory activity of shiitake extract decreased from 100% to 51.99% in the tested concentration range (shown in Figure 2a), while the reference inhibitor (N‐methoxysuccinyl‐Ala‐Ala‐Pro‐Val‐chloromethyl ketone) decreased from 100% to 4.79% (shown in Figure 2b). These findings indicate that shiitake extract exhibits a measurable elastase inhibitory activity, demonstrating inhibition within the tested concentration range when evaluated alongside a reference inhibitor.

### 3.3 Collagenase Inhibition

**FIGURE 3:**
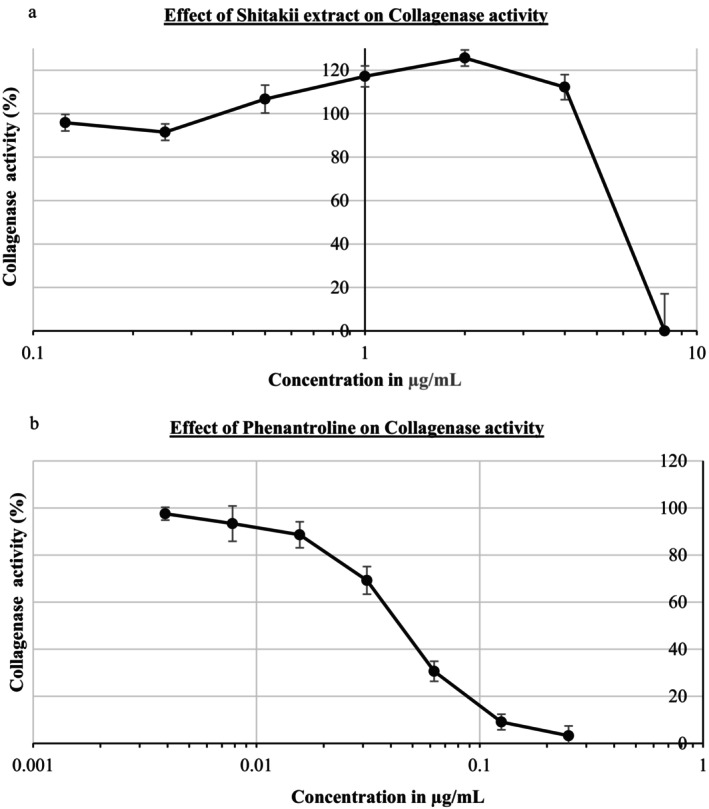
Collagenase activity of Shiitake extract (a), and reference molecule (b). Collagenase activity is expressed as a percentage of the control (0 concentration), which was set to 100%. Data are presented as mean ± standard deviation (SD) of triplicate experiments (*n* = 3). Values below zero were set to zero, as they reflect the absence of detectable collagenase activity due to background signal overcorrection following blank subtraction, indicating complete inhibition within the sensitivity limits of the assay.

The collagenase inhibitory activity of shiitake extract was evaluated using phenanthroline as a reference inhibitor. The reference compound exhibited a strong inhibitory effect across the tested concentration range, reducing collagenase activity from 100% to 3.25% (Figure 3b). In contrast, shiitake extract caused a concentration‐dependent reduction in collagenase activity, decreasing from 100% to levels below the detection limit of the assay at the highest tested concentration (Figure 3a), indicating a complete suppression of measurable enzyme activity.

### 3.4 Tyrosinase Inhibiton

**FIGURE 4:**
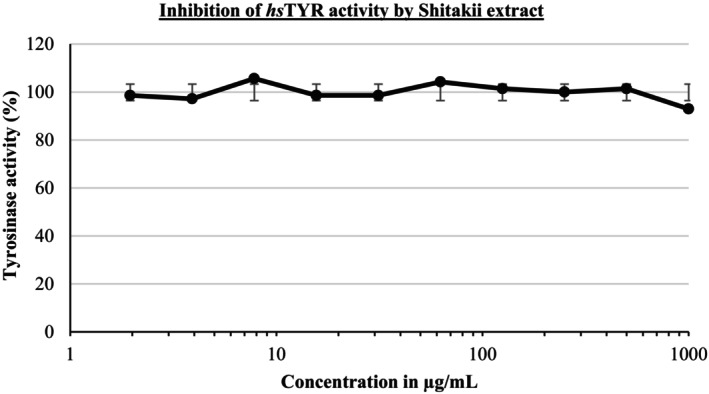
Tyrosinase activity of shiitake extract.

The effect of shiitake extract on human tyrosinase (*hs*TYR) activity was evaluated at concentrations ranging from 1 to 1000 μg/mL. As seen in Figure 4, the enzyme activity remained almost unchanged, with values fluctuating around 100%. These results indicate that shiitake extract did not exhibit a significant inhibitory effect on tyrosinase under the tested conditions. As no measurable tyrosinase inhibition was observed, the results are presented to reflect the absence of inhibitory activity under the tested conditions.

### 3.5 Cytotoxicity and Determination of Dose Limit

**FIGURE 5:**
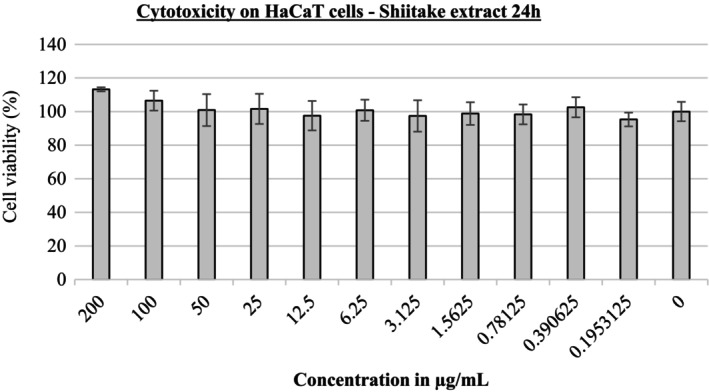
Cytotoxicity of shiitake extract.

The cytotoxicity and dose–response relationship of shiitake extract was evaluated on HaCaT human keratinocytes after 24 h of exposure (shown in Figure 5). The results showed that the extract did not produce significant cytotoxic effects in the concentration range tested (0.19–200 μg/mL). Cell viability values remained above 90% for all concentrations, indicating that the extract was well tolerated by keratinocytes. Accordingly, no concentration‐dependent decrease in viability was observed, and the IC₅₀ value could not be determined in the tested range. These findings indicate that the shiitake extract is non‐cytotoxic and biocompatible.

### 3.6 Preparation and Characterization of a Topical Skin Cream Containing Shiitake Extract

**FIGURE 6:**
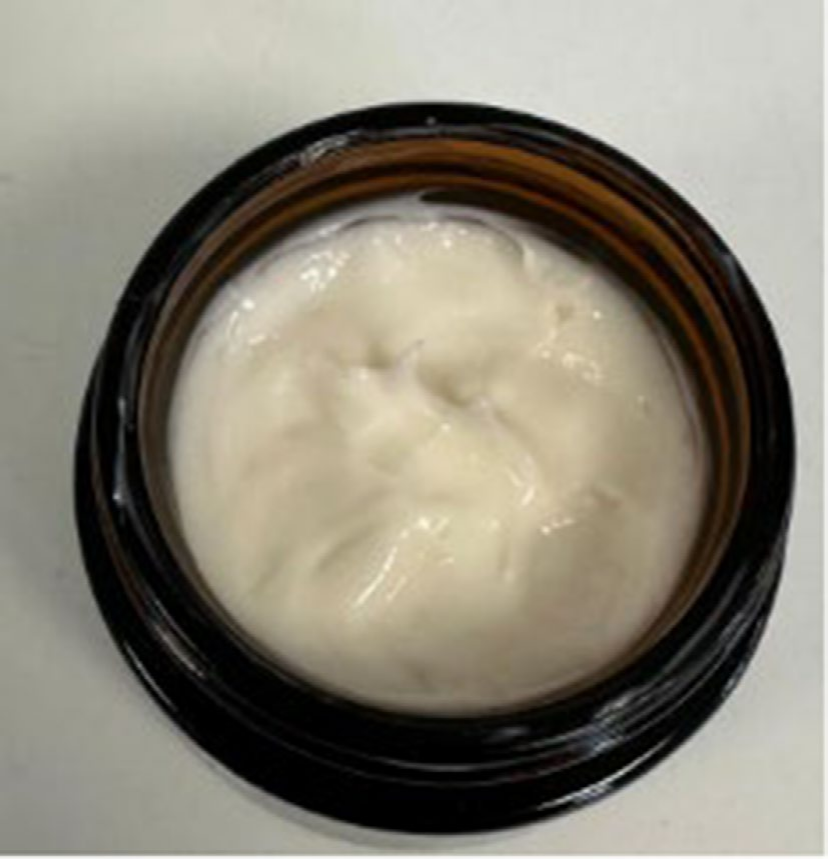
Topical shiitake extract cream.

The obtained shiitake extract‐based topical cream is white, as shown in Figure 6. The fragrance was designed with ylang‐ylang and cedar essential oils to mask the mushroom odor, giving it a herbal scent. The pH, density, and viscosity values of the formulas are shown in Table 2. The pH value is approximately 5.5, which is compatible with skin pH. The log viscosity value of the resulting cream was found to be 1.40.

**TABLE 2:** Physicochemical properties of topical cream.

Formulation	pH ± SD	Density ± SD (g/mL)	Viscosity ± SD (cP)	Log viscosity
Cream	5.496 ± 0.011	1.213 ± 0.007	25.118 ± 0.350	1.40

### 3.7 Stability Analysis of the Topical Cream

The topical cream obtained in this study was aged in a stability cabinet, and controls were performed at week 1, month 1, month 2, and month 3. As shown in Table 3, there was no color/odor change, pH change, or packaging change in the topical cream after aging. Furthermore, no phase separation or microbiological growth was observed in the cream.

**TABLE 3:** Stability analysis of topical cream.

Parameters	1. Week	1. Month	2. Month	3. Month
	28.06.2025	22.07.2025	22.08.2025	22.09.2025
Color	White	White	White	White
Odor	Herbal	Herbal	Herbal	Herbal
Appearance	No change	No change	No change	No change
pH	5.5	5.53	5.58	5.55
Package	No change	No change	No change	No change
Phase separation	No change	No change	No change	No change
Microbiological growth	No	No	No	No

## 4 Discussion

There are studies suggesting that mushrooms are a potent source of dietary antioxidants, reduce oxidative stress, and prevent disease. A study comparing the antioxidant activity of five different laboratory‐produced mushroom extracts (chaga, maitake, reishi, shiitake, and turkey tail) found that shiitake had antioxidant activity equivalent to 120 μM Trolox (TE), supporting our findings [28]. In a study investigating the effect of drying on the amino acid composition, phenolic compounds, and antioxidant activity of shiitake mushroom extract, the antioxidant activity of shiitake mushroom extract after drying was found to be 65.01 to 81.39 μM Trolox (TE) [29]. In this study, heat was applied for 20 days during drying, whereas in our study, the antioxidant values were thought to be higher because freeze‐drying was used. Like the shiitake extracts obtained by acetonic, methanolic and hot water extraction methods, the extract obtained by the ethanolic extraction method used in our study also has high antioxidant activity values, and carotenoids, polyphenols, vitamins, polysaccharides and minerals are thought to be the main source of antioxidant effect [30]. The antioxidant activity of the shiitake extract was evaluated using total antioxidant capacity (TAC) as a preliminary screening assay. While the obtained TAC values were moderate compared to reference antioxidants, antioxidant activity was considered a supportive parameter in this study, with the primary focus placed on enzyme inhibitory activity and formulation performance.

Human skin consists of the epidermis, which is connected and supported by connective tissue to the underlying dermis. The epidermis is the skin's protective layer and responsible for skin renewal, while the dermis is responsible for skin durability and aging. The dermis contains elastic connective tissue for skin elasticity and collagen for its strength, producing elastin and collagen [23]. Elastin is an ECM protein that provides elasticity to connective tissues such as the aorta, lungs, cartilage, elastic ligaments, and skin. Elastase is a metalloproteinase enzyme that can degrade elastin. Chronic exposure to elastase leads to damage to elastic fibers, resulting in reduced skin elasticity and wrinkles. Similarly, collagen is a major component of the skin's extracellular matrix, responsible for skin elasticity, resilience, and strength. Collagenase is a therapeutic enzyme responsible for breaking down the peptide bonds in collagen [31]. Since elastase and collagenase activity increases significantly with age, it is extremely important for cosmetic ingredients to inhibit elastase and collagenase in order to delay skin aging and reduce wrinkles [32]. While studies on the anti‐elastase and anti‐collagenase activities of different mushroom species are available in the literature, there are very few reports on the anti‐elastase and anti‐collagenase activities of shiitake mushroom extracts or their metabolites. One study reported the anti‐collagenase and anti‐elastase activities of *T. matsutake* extract after treating dermal fibroblast cells at concentrations of 1–100 g/mL for 72 h. It reduced elastase activity by up to 81.4% in a dose‐independent manner, while it had no effect on collagenase enzyme [23]. In another study, the antioxidant, anti‐tyrosinase and anti‐collagenase activity of *Grifola fondosa* mycelial extract was investigated and it significantly inhibited collagenase by 20% and 40% [33]. In another study, an exopolysaccharide was isolated from submerged mycelial culture of *Grifola frondosa* and its collagenase enzyme inhibition ability in dermal fibroblasts after UVA exposure was found to be 61.1% [34]. This study evaluated the effects of shiitake mushroom extract on elastase and collagenase activities. The extract exhibited a marked inhibitory effect on elastase activity, achieving an inhibition of 51.99%. Regarding collagenase, the shiitake extract induced a concentration‐dependent suppression of enzymatic activity, reducing measurable collagenase activity to levels below the detection limit of the assay at the highest tested concentration. This inhibitory profile was comparable to that of phenanthroline, a well‐established synthetic collagenase inhibitor. Overall, these findings indicate that ethanolic shiitake extract possesses significant elastase and collagenase inhibitory activities, supporting its potential as a bioactive ingredient for anti‐aging pharmacological and dermatological applications. Additionally, the observed enzyme inhibitory effects may contribute to the previously reported nutraceutical relevance of shiitake‐derived bioactive compounds.

In such naturally sourced products, the toxic effect on the skin and the dosage are very important. The shiitake extract obtained in this study did not show any cytotoxic effects on HaCaT human keratinocytes after 24 h of exposure at concentrations up to 200 μg/mL, and the cells maintained their viability. In a 2019 study, ethanolic extract of the shiitake mushroom did not show cytotoxicity in the HaCaT cell line at concentrations up to 100 μg/mL, which is consistent with the results of our study [35]. However, other studies have shown a significant decrease in cell viability at 1 μg/mL, with a decrease of up to 30% at the highest concentration tested (10 μg/mL) [36, 37]. These results suggest that higher concentrations of the extract may be harmful to skin cells and that a maximum use of 1% in dermocosmetic products would be safe.

A topical cream with suitable physicochemical and application characteristics was developed by incorporating the shiitake extract into the water phase due to its water solubility. The formulation and ingredient selection ensured a skin‐compatible pH. Stability tests demonstrated that no phase separation occurred during an accelerated two‐year shelf‐life simulation, no microbial growth was observed, and the cream retained its physicochemical properties throughout storage. Shiitake extract, widely studied as a nutraceutical and commercially available, represents a promising dermocosmetic active. It is naturally derived, non‐toxic, and exhibits bioactivity relevant to anti‐aging applications. However, as the present findings are based on in vitro enzyme inhibition assays and formulation characterization, this study should be regarded as a preliminary investigation. Further studies focusing on skin‐related functional performance, including cellular‐based evaluations of collagen biosynthesis, release behavior, skin penetration, and in vivo or clinical validation, are required to fully substantiate the dermocosmetic efficacy of shiitake‐based formulations.

## Author Contributions

In this study, S.D.K. contributed to the study planning, procurement of necessary materials, design of the shiitake extract topical cream formulation, performance and interpretation of physicochemical and stability analyses of the topical cream, literature review, writing of the article, and content control. P.J., A.G., and G.C. contributed to the antioxidant activity, enzyme inhibition, and cytotoxicity analyses of the shiitake extract. R.D. contributed to the provision of infrastructure and interpretation of the analysis results. Finally, B.K. performed the extraction and freeze‐drying of the dried shiitake extract.

## Ethics Statement

Ethical approval was not required for this study as it did not involve human participants, animal experiments, or the use of cell lines. The research was conducted in accordance with institutional and national ethical standards.

## Consent

The authors have nothing to report.

## Conflicts of Interest

The authors declare no conflicts of interest.

## Data Availability

The data that support the findings of this study are available from the corresponding author upon reasonable request.
